# The Effect of Gut Microbiota on the Progression of Intervertebral Disc Degeneration

**DOI:** 10.1111/os.13626

**Published:** 2023-01-04

**Authors:** Bo Yao, Youquan Cai, Weiguo Wang, Jia Deng, Lei Zhao, Ziwei Han, Li Wan

**Affiliations:** ^1^ Department of Spine Surgery Want want hospital Changsha Hunan China; ^2^ Department of Spine Surgery, the Third Xiangya Hospital Central South University Changsha Hunan China

**Keywords:** Autophagy, Fecal Microbiota Transplantation, Gut Microbiota, Inflammation, Intervertebral Disc Degeneration

## Abstract

**Objective:**

Intervertebral disc degeneration (IDD) is the main cause of back pain, and its treatment is a serious socio‐economic burden. The safety and treatment of fecal microbiota transplantation (FMT) has been established. However, the relationship between FMT and IDD still unclear. We aimed to explore whether FMT plays a role in IDD to provide a reference for the treatment of IDD.

**Methods:**

An experimental model of IDD was established using 2‐month‐old male Sprague–Dawley rats. FMT was performed by intragastric gavage of IDD rats with a fecal bacterial solution. Rat serum, feces, and vertebral disc tissue were collected after surgery for 2 months. The levels of TNF‐α, IL‐1β, IL‐6, matrix metalloproteinase (MMP)‐3, MMP‐13, Collagen II, and aggrecan in the serum or vertebral disc tissue were measured by an enzyme‐linked immunosorbent assay, immunohistochemistry, quantitative real‐time polymerase chain reaction, or western blotting. We also examined the pathology of the vertebral disc tissue using hematoxylin and eosin (HE) and safranin O‐fast green staining. Finally, we examined the gut microbiota in rat feces using 16 S rRNA gene sequencing.

**Results:**

We found that the expression of TNF‐α, IL‐1β, IL‐6, MMP‐3, MMP‐13, NLRP3 and Caspase‐1 increased in the IDD group rats. In contrast, Collagen II and aggrecan levels were downregulated. Additionally, vertebral disc tissue was severely damaged in the IDD group, with disordered cell arrangement and uneven safranin coloration. FMT reversed the effects of IDD modeling on these factors and alleviated cartilage tissue damage. In addition, FMT increased the gut microbiota diversity and microbial abundance in rats treated with IDD.

**Conclusion:**

Our findings suggest that FMT has a positive effect in maintaining cellular stability in the vertebral disc and alleviating histopathological damage. It affects the diversity and abundance of gut microbiota in rats with IDD. Therefore, FMT may serve as a promising target for amelioration of IDD.

## Graphical abstract

Fecal microbiota transplantation (FMT) inhibited TNF‐α, IL‐1β, IL‐6, MMP‐3, MMP‐13, NLRP3, and Caspase‐1 levels, up‐regulated collagen II and aggrecan expression, restored gut microbiota homeostasis, thereby improving intervertebral disc degeneration in mice.

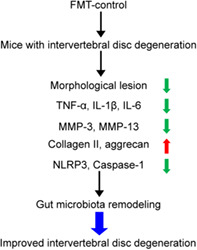

## Introduction

Low back pain (LBP) is closely related to intervertebral disc degeneration (IDD).^1^, ^2^ The exact pathogenesis of IDD is believed to be the result of changes in the microenvironment within *in vitro* diagnostics caused by multiple factors.^3^, ^4^ The main strategy for all IDD treatments is to prevent cell loss due to programmed or regulated cell death.^5^

The intervertebral disc is located between the vertebrae,^6^ with gelatinous nucleus pulposus (NPs) as the central structure. NPs are surrounded by a fibrous plate annulus (AF)^7^ and sandwiched between the upper and lower cartilage endplates (CEPs).^8^, ^9^ NPs play a role in maintaining homeostasis by secreting the extracellular matrix (ECM). A homeostatic imbalance between ECM anabolism and catabolism results in ECM reduction.^8^, ^9^ IDD is accompanied by changes in ECM homeostasis. Increased levels of secreted proinflammatory cytokines further promote ECM degradation and changes in diagnostic cell phenotypes. This results in vertebral disc protrusion and radicular pain.^10^ In addition to changes in ECM homeostasis, IDD is associated with cellular inflammation and apoptosis.^11^, ^12^, ^13^, ^14^ Some studies have shown that cells die in the form of pyroptosis after inflammasome formation.^15^, ^16^ Pyroptosis releases large amounts of interleukin‐ (IL) 1β and IL‐18, leading to further inflammatory damage.^16^

Gut microbiota also affects intervertebral disc homeostasis.^17^, ^18^ There were three potential ways of influence this: (1) the bacteria crossed the intestinal epithelial barrier into the intervertebral disc; (2) Bacteria regulate the mucosal and systemic immune system; (3) Bacteria regulate the metabolism of substances in the intestinal epithelium.^19^ Clinical testing revealed nearly 60 overlapping bacterial strains between the intervertebral disc and the intestine. There are differences in biodiversity and abundance between healthy and diseased discs. Protective bacteria are abundant in normal discs, whereas putative pathogens are present in IDDs.^20^ Therefore, regulation of the intestinal microbiota may affect the diversity and abundance of the microbiota in the intervertebral disc to regulate IDD.

Fecal microbiota transplantation (FMT) is associated with inflammation regulation and bacterial flora homeostasis *in vivo*. FMT is a therapeutic approach for colonizing the patient's gut with fecal suspension from a healthy individual.^21^ Altering the composition of the host gut microbiota by fecal microbiota transplantation has been suggested as a potential novel strategy for regulating bone homeostasis.^22^ Maternal FMT can restore the gut microbiota of infants born by cesarean section (CS) postpartum.^23^ FMT has been shown to have favorable therapeutic effects in patients with severe antibiotic‐associated diarrhea (AAD) without infectious complications.^24^ Researchers have provided limited evidence of a link between modic changes (a type of vertical disc degeneration) and gut microbiota by 16 S sequencing.^25^ However, whether FMT has a therapeutic effect on IDD has not been further investigated. As the efficacy of FMT in related diseases has been established, we aimed to explore its potential role in IDD. The objectives of our study were as follows: (i) Clarify the effects of FMT on histopathological damage and intestinal flora in IDD rats; and (ii) Explore the regulation of FMT on ECM homeostasis, inflammation and pyrogenesis.

## Methods

### Animals Model

The experimental animals were Sprague–Dawley (SD) rats (2‐months‐old, male). The rats were purchased from Hunan SJA Laboratory Animal Co., Ltd. (Changsha, China). Rats were randomly divided into three groups: control, IDD, and IDD + FMT. Rats were anesthetized with 2% pentobarbital (40 mg/kg). Grades 7–8 (Co7‐8) and 8–9 (Co8‐9) of the rat coccyx were punctured from the back with a 20‐gauge needle.^26^, ^27^, ^28^ After the needle was completely inserted into the full thickness of the annulus fibrosus, it was rotated for 5 s and held for 30 s to ensure the effect of degenerative damage.

We collected 10 g of fresh feces from 2‐month‐old male rats. Fifty milliliters of sterile physiological saline was added and stirred at 37°C. The fecal solution was filtered through a double layer of sterile gauze. The filtered fecal residue was suspended in 100 mL of sterile physiological saline to obtain a fecal bacterial solution. Rats in the IDD + FMT group were intragastrically administered (5 mL/kg) by gavage once a day for 14 days (from the second day after the IDD operation).^29^, ^30^ The control and IDD groups were gavaged daily with the same volume of sterile physiological saline. Feces were collected 2 months after the operation, and the rats were sacrificed for subsequent analysis and detection.

### Enzyme‐Linked Immunosorbent Assay

We determined the levels of TNF‐α, IL‐1β, IL‐6, matrix metalloproteinase (MMP)‐3, MMP‐13, Collagen II, and aggrecan in the serum and vertebral disc tissue. We used TNF‐α (RTA00, Bio‐Techne, Minneapolis, Minnesota, USA), IL‐1β (RLB00, Bio‐Techne, Minneapolis, Minnesota, USA), IL‐6 (437107, BioLegend, San Diego, CA, USA), MMP‐3 (SEA101Ra, Cloud‐Clone Corp, Houston, TX, USA), MMP‐13 (SEA099Ra, Cloud‐Clone Corp, Houston, TX, USA), Collagen II (ER0852, Wuhan Fine Biotech, Wuhan, China), and aggrecan (SEB908Ra, Cloud‐Clone Corp, Houston, TX, USA) Enzyme‐Linked Immunosorbent Assay (ELISA) kits to measure relative factors.

### Immunohistochemical

Immunohistochemical (IHC) was used to measure the distribution of NLRP3, Caspase‐1, MMP‐3, and Collagen II in vertebral disc tissue. After dehydration, the sections were placed in 0.01 M citrate buffer (pH 6.0) and kept at a high temperature for 20 min. After the sections were cooled and washed, 1% periodic acid was used for 10 min to eliminate endogenous enzymes. Next, NLRP3 primary antibody (PA5‐79740, 1:400, Cell Signaling Technology, Danvers, MA, USA), Caspase‐1 primary antibody (22915‐1‐AP, 1:100, Proteintech, Chicago, IL, USA), MMP‐3 primary antibody (17873‐1‐AP, 1:100, Chicago, IL, Proteintech, USA), and Collagen II primary antibody (bs‐10589R, 1:200, Bioss, Beijing, China) were incubated. The secondary antibodies were then added for incubation. Subsequently, the sections were cultured with 3,3′‐diaminobenzidine (DAB), hematoxylin, and alcohol dehydration. Finally, the sections were blocked with a neutral resin and examined under a microscope (BA410T; Motic, Hongkong, China).

### Hematoxylin and Eosin Staining

We performed a pathological examination of the vertebral disc tissue with hematoxylin and eosin (HE). Tissue sections were baked at 60°C, soaked in xylene, and dehydrated in alcohol. The sections were stained with hematoxylin for 5 min and stained with eosin for 5 min. After washing with distilled water, the sections were dehydrated in an alcohol gradient. Finally, the sections were blocked with neutral resin and examined under a microscope (BA410T; Motic, Hongkong, China).

### Safranin O‐Fast Green (S‐O)

We also examined the pathology of the vertebral disc tissue with S‐O. Tissue sections were baked at 60°C, soaked in xylene, and dehydrated in alcohol. Sections were stained with fast green stain for 5 min and then stained with safranin O solution again for 30 s. Sections were dehydrated after washing with distilled water. Finally, the sections were blocked with neutral resin and examined under a microscope (BA410T; Motic, Hongkong, China).

### 16 S rRNA Gene Sequencing

First, we extracted fecal DNA using a genomic DNA extraction kit (dp328‐02, Tiangen, Beijing, China). Next, bacterial primers for the V3‐V4 region (341F:5’‐CCTACGGGNGGCWGCAG‐3′ and 805R:5’‐GACTACHVGGGTATCTAATCC‐3′) and the fusion enzyme (K1031, APExBIO, Houston, TX, USA) were used for PCR amplification. Raw data were obtained using an Illumina Novaseq6000 PE250. The Qiime 2 (2020.2) analysis process was used to call DADA2 for quality control in order to obtain useful clean data. Each amplicon sequence variant/OTU sequence was annotated using the silvA‐132‐99 database to obtain information on the corresponding species. The alpha diversity index of each sample was calculated using Qiime 2 software. The distance matrix was obtained based on the Bray–Curtis algorithm, which was used for beta diversity analysis and visualization.

### Quantitative Real‐Time Polymerase Chain Reaction

We examined the expression of TNF‐α, IL‐1β, IL‐6, MMP‐3, MMP‐13, Collagen II, aggrecan, NLRP3, and Caspase‐1 in vertebral disc tissue. First, total RNA was extracted using TRIzol Reagent (15596–026, Invitrogen, Grand Island, NY, USA). Next, mRNA was reverse‐transcribed to cDNA using an mRNA reverse transcription kit (CW2569, Kangwei Century, Beijing, China). Primers for TNF‐α, IL‐1β, IL‐6, MMP‐3, MMP‐13, Collagen II, aggrecan, NLRP3, Caspase‐1, and β‐actin were provided by Sangon Biotech (Sangon Biotech, Shanghai, China) (Table 1). β‐actin was used as a reference. DNA amplification and detection were performed using a fluorescent quantitative PCR instrument (PIKOREAL96, Thermo Fisher, Waltham, MA, USA). 2^−△△CT^ was used to assess relative mRNA levels.

**TABLE 1:** The primer sequence

Primer ID	5′‐3′	Product length (bp)
β‐Actin	**F:** ACATCCGTAAAGACCTCTATGCC	223
	**R:** TACTCCTGCTTGCTGATCCAC	
TNF‐α	**F:** CCCCTCTATTTATAATTGCACCT	167
	**R:** CTGGTAGTTTAGCTCCGTTT	
IL‐1β	**F:** CAGCAGCATCTCGACAAGAG	123
	**R:** AAAGAAGGTGCTTGGGTCCT	
IL‐6	**F:** TCACTATGAGGTCTACTCGG	141
	**R:** CATATTGCCAGTTCTTCGTA	
MMP‐3	**F:** CCTCTGAGTCTTTTCATGGAGGG	234
	**R:** TGTCTGTAGCCCAGGAGTGT	
MMP‐13	**F:** CAAAGACTATCCCCGCCTCA	147
	**R:** ACTCTCACAATGCGATTACTCC	
CollagenII	**F:** ACCCTCAACCCCAAAACAACACA	100
	**R:** TCAGGTCAGCCATTCAGTGC	
aggrecan	**F:** ACAGACACCCCTACCCTTGC	193
	**R:** CCTCACATTGCTCCTGGTCGAT	
NLRP3	**F:** CACCTCTTCTCTGCCTACCTG	181
	**R:** AGCTGTAAAATCTCTCGCAGT	
Caspase‐1	**F:** CTAGACTACAGATGCCAACCAC	128
	**R:** GGCTTCTTATTGGCATGATTCCC	

### Western Blot

We also measured the levels of TNF‐α, IL‐1β, IL‐6, MMP‐3, MMP‐13, Collagen II, aggrecan, NLRP3, and Caspase‐1 in vertebral disc tissue. First, the vertebral disc tissue was fully ground in RIPA Lysate (AWB0136; Abiowell, Changsha, China). Subsequently, the grinding fluid was centrifuged, and the supernatant was collected. The protein content was measured using a BCA protein kit (ab102536, Abcam, Cambridge, UK).

Based on the protein quantification results, the markers and denatured proteins were dotted into the wells. Electrophoresis was performed at a constant voltage of 75 V for 130 min. Electrophoresis was terminated when bromophenol blue was electrophoresed at the bottom of the gel. After the transfer, the membrane was removed and washed once with 1 × PBST. Subsequently, we disposed the membrane for 1.5 h under PBST (5% skimmed milk) powder. Next, we added primary and secondary antibodies. A chemiluminescence imaging system (ChemiScope6100, Clinx, Shanghai, China) was used to calculate the protein samples on the membrane. β‐actin was used as an internal reference. Antibody information is presented in Table 2.

**TABLE 2:** The information of antibody

Name	Article number	Source	Dilution rate	Molecular weight	Transfer film time	Company
TNF‐α	60,291‐1‐Ig	Mouse	1:5000	17–22 KDa	40 min	Proteintech (Chicago, IL, USA)
IL‐1β	ab254360	Rabbit	1:1000	30 KDa	50 min	Abcam (Cambridge, UK)
IL‐6	21,865‐1‐AP	Rabbit	1:500	20 KDa	40 min	Proteintech (Chicago, IL, USA)
MMP‐3	17,873‐1‐AP	Rabbit	1:1000	45–47 KDa	70 min	Proteintech (Chicago, IL, USA)
MMP‐13	18,165‐1‐AP	Rabbit	1:1000	65–70 KDa	90 min	Proteintech (Chicago, IL, USA)
CollagenII	ab188570	Rabbit	1:5000	141 KDa	150 min	Abcam (Cambridge, UK)
aggrecan	ab36861	Rabbit	1 μg/mL	110 KDa	130 min	Abcam (Cambridge, UK)
NLRP3	ab263899	Rabbit	1:1000	118 KDa	150 min	Abcam (Cambridge, UK)
Caspase‐1	ab179515	Rabbit	1:1000	45/10/12 KDa	75 min	Abcam (Cambridge, UK)
β‐Actin	66,009‐1‐Ig	Mouse	1:5000	42 KDa	60 min	Proteintech (Chicago, IL, USA)
HRP goat anti‐Rabbit IgG	SA00001‐2	Rabbit	1:6000	/	90 min	Proteintech (Chicago, IL, USA)
HRP goat anti‐mouse IgG	SA00001‐1	Mouse	1:5000	/	90 min	Proteintech (Chicago, IL, USA)

### Statistical Analysis

Graphpad Priam (version 9.0; GraphPad Software, La Jolla, CA, USA) was used for statistical analysis. Kolmogorov–Smirnov test was used to analyze whether the data conformed to a normal distribution. The normally distributed continuous variables were represented as the mean ± standard deviation (X̅ ± SD). One‐way analysis of variance (ANOVA) was used to compare the data differences between multiple groups. *P*‐Value <0.05 was considered statistically significant.

## Results

### Histopathological Analysis of Vertebral Disc after FMT

**Fig. 1:**
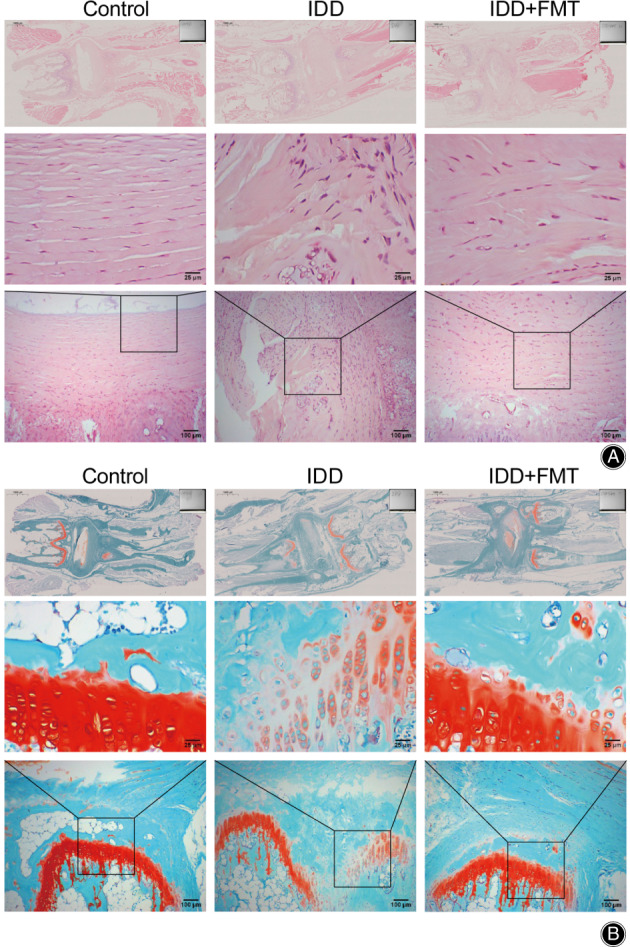
Histopathological analysis of vertebral disc after FMT. (A) A micrograph showing HE staining of rat intervertebral disc tissue. (Scale bar = 25/100/1000 μm). (B) A micrograph showing S‐O staining of rat intervertebral disc tissue. (Scale bar = 25/100/1000 μm). FMT, fecal microbiota transplantation.

First, we analyzed the histopathological findings of the vertebral discs. The cells in the vertebral disc tissue of the control group were normal and regular, and no lesions were observed. In the IDD group, the number of cells decreased, and the arrangement was disordered. The IDD + FMT group showed a therapeutic effect, and tissue damage was effectively alleviated (Figure 1A). The S‐O results showed the same tissue damage trend as the HE results. No damage was observed in the control group, and safranine coloration was uniform. The vertebral disc tissue in the IDD group was severely damaged, and safranin coloration was uneven. The treatment effect was observed in the IDD + FMT group, and tissue damage was observed between the control and IDD groups (Figure 1B). Therefore, FMT alleviated histopathological damage caused by IDD.

### FMT Changed the Levels of Inflammatory Factors in Rats

**Fig. 2:**
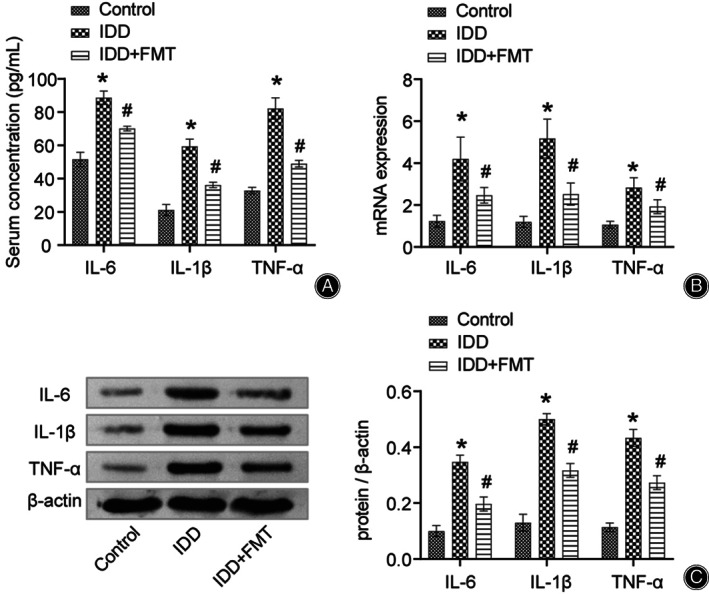
FMT alters inflammatory factor levels in rats. (A) Quantification of TNF‐α, IL‐1β, and IL‐6 in the rat serum. (B) Expression of TNF‐α, IL‐1β, and IL‐6 in rat intervertebral disc tissues. (C) Protein expression of TNF‐α, IL‐1β, and IL‐6 in the rat intervertesbral disc tissue. *P < 0.05 vs. control. #*P* < 0.05 vs. IDD. FMT, fecal microbiota transplantation; IDD, intervertebral disc degeneration.

To investigate whether FMT affects inflammatory factors, we first assessed the expression of TNF‐α, IL‐1β, and IL‐6 in the rat serum and vertebral disc tissue. ELISA, quantitative real‐time polymerase chain reaction (qRT‐PCR), and western blotting showed that TNF‐α, IL‐1β, and IL‐6 were upregulated in the IDD group compared to the control group (Figure 2A‐C). FMT reversed the effects of IDD modeling on inflammatory factors and significantly downregulated their levels (Figure 2A‐C). Therefore, FMT led to the downregulation of inflammatory factors in IDD rats.

### FMT Altered Catabolic Enzyme (Matrix Metalloproteinase, MMP) and Extracellular Matrix (Collagen II and Aggrecan) Levels in the Vertebral Disc Tissue

**Fig. 3:**
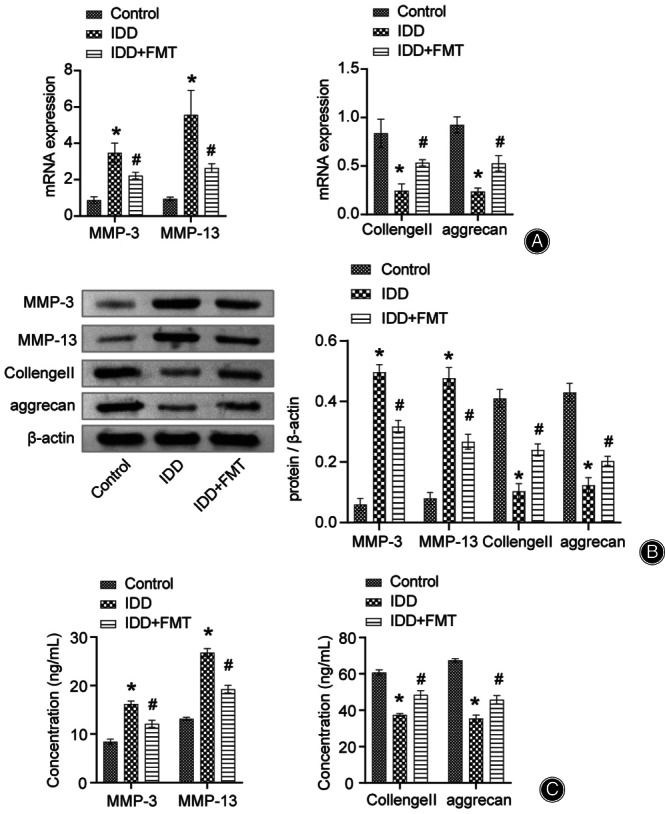
FMT altered catabolic enzymes and extracellular matrix levels in vertebral disc tissue. (A) qRT‐PCR data representing the expression of MMP‐3, MMP‐13, Collagen II, and aggrecan in rat intervertebral disc tissue. (B) Western blot data representing the protein expression of MMP‐3, MMP‐13, Collagen II, and aggrecan in rat intervertebral disc tissue. (C) ELISA data representing the quantification of MMP‐3, MMP‐13, Collagen II, and aggrecan in rat serum. *P < 0.05 vs. control. #*P* < 0.05 vs. IDD. FMT, fecal microbiota transplantation; IDD, intervertebral disc degeneration; qRT‐PCR, quantitative real‐time polymerase chain reaction; MMP, matrix metalloproteinase.

Imbalance of the extracellular matrix in the vertebral disc tissue leads to problems such as abnormal stress in the vertebral disc and eventually causes IDD. Therefore, we investigated the effects of FMT on vertebral disc ECM homeostasis. We found that the levels of MMP‐3 and MMP‐13 in the IDD group were significantly upregulated by qRT‐PCR (Figure 3A). After FMT treatment, the levels of MMP‐3 and MMP‐13 decreased (Figure 3A). The changing trend of Collagen II and aggrecan was always opposite to that of MMP (Figure 3A). Additionally, we detected the above factors in the vertebral disc tissue by western blotting and ELISA (Figure 3B,C, respectively). The detection results were consistent with those of qRT‐PCR. Thus, FMT positively affects the maintenance of vertebral disc extracellular matrix homeostasis.

### FMT Changed the Level of Pyroptosis Factor in Vertebral Disc Tissue

**Fig. 4:**
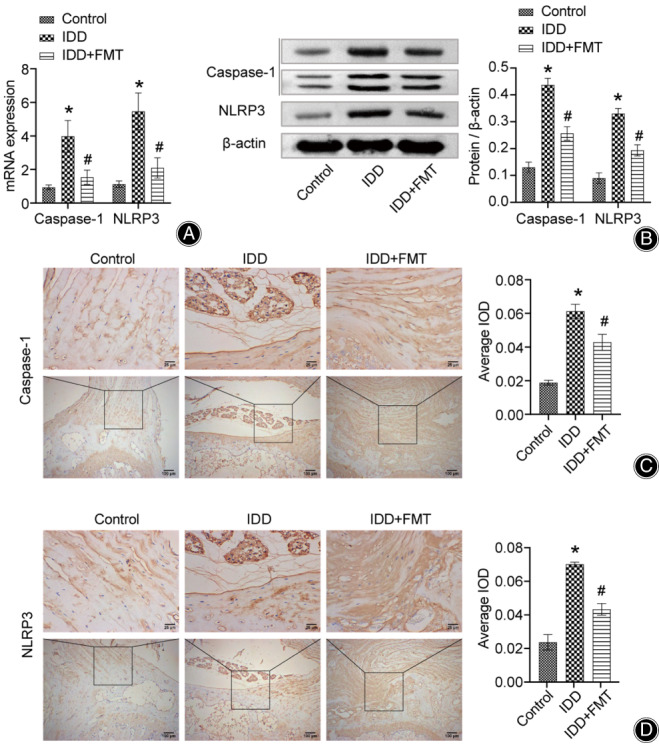
FMT changed the level of pyroptosis in vertebral disc tissue. (A) Expression of Caspase‐1 and NLRP3 in rat intervertebral disc tissues. (B) Protein expression of Caspase‐1 and NLRP3 in rat intervertebral disc tissue. (C) Micrograph showing IHC staining for Caspase‐1 in rat intervertebral disc tissue. (Scale bar = 25/100 μm). (D) Micrograph showing IHC staining of NLRP3 in rat intervertebral disc tissues. (Scale bar = 25/100 μm). *P < 0.05 vs. control. #*P* < 0.05 vs. IDD. FMT, fecal microbiota transplantation; IDD, intervertebral disc degeneration; IHC, immunohistochemical.

Next, the influence of FMT on vertebral disc tissue pyroptosis factor was examined. We assessed changes in pyroptotic factors (NLRP3 and Caspase‐1) in vertebral disc tissues using qRT‐PCR, western blotting, and IHC. The changes in the levels of pyroptosis factors in the three assays were consistent (Figure 4A‐D). IDD modeling experiments upregulated the expression of NLRP3 and Caspase‐1. However, FMT alleviated the modeling damage and significantly downregulated the levels of these factors. Therefore, FMT also has a positive effect on the alleviation of pyroptosis in IDD.

### Changes in Gut Microbiota after FMT

**Fig. 5:**
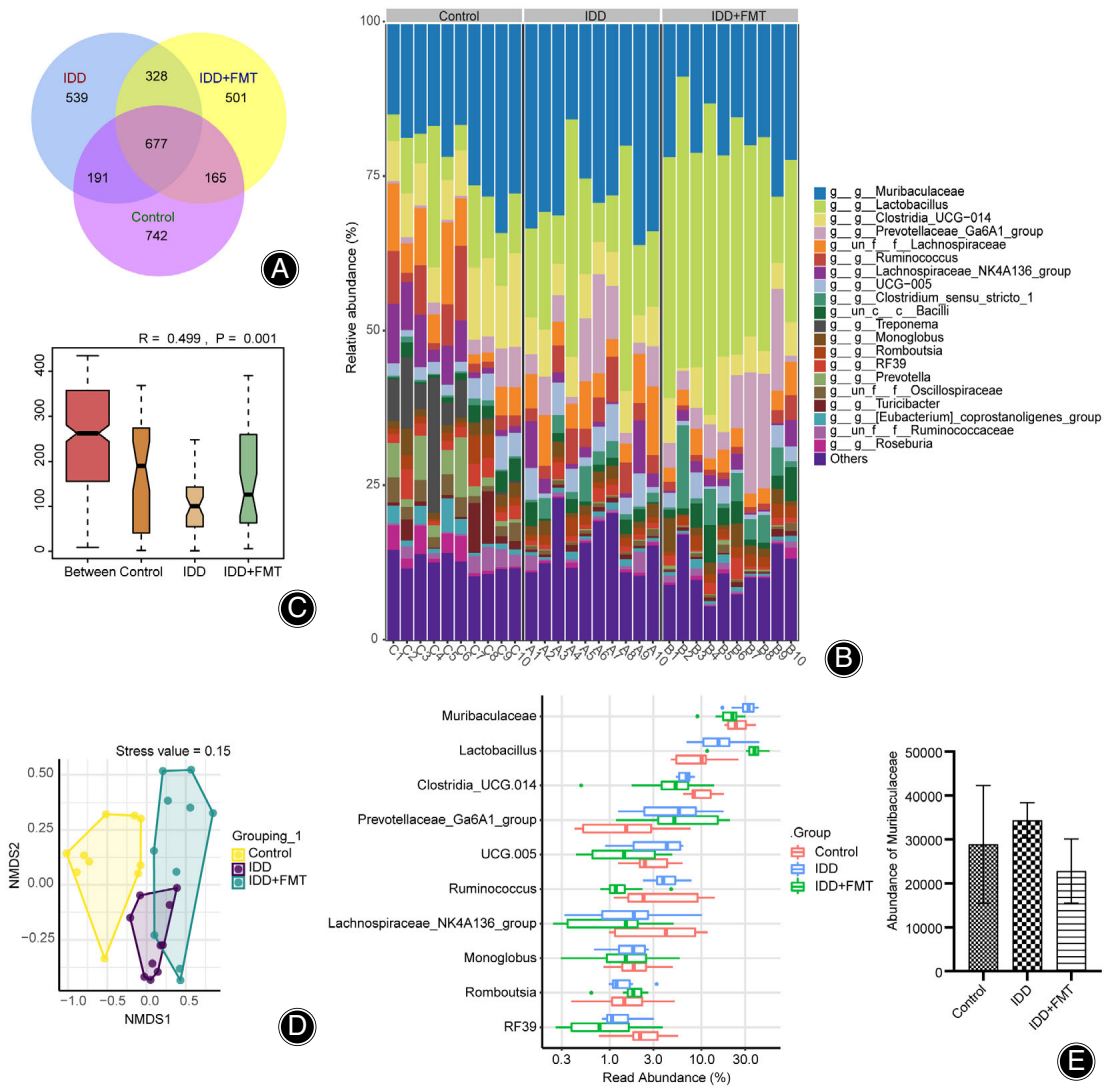
Changes in gut microbiota after FMT. (A) Venn diagram. The number on the petal represents the number of ASVs unique to the sample. (B) Histogram of the relative abundance of top 20 ASVs at the genus level. (C) Anosim analyses. This was used to analyze differences between groups. (D) NMDS index shows the differences between groups. E. Read abundance of top 10 dominant flora at the genus level. FMT, fecal microbiota transplantation; ASV, amplicon sequence variant; NMDS, non‐metric multidimensional analysis.

Finally, we performed 16 S rRNA gene sequencing of rat feces. First, we used a Venn diagram to visualize the common amplicon sequence variants (ASVs) in both groups (Figure 2A). We found 742 ASVs specific to the control, 539 ASVs specific to the IDD, 501 ASVs specific to the IDD + FMT, 868 ASVs common to the controls and IDD, and 1005 ASVs common to the IDD and IDD + FMT (Figure 5A). The histogram shows that the genus level microbes mainly included *Muribaculaceae, Lactobacillus, Clostridia_UCG‐014, Prevotellaceae Ga6A1 group, Lachnospiraceae, Ruminococcus, Lachnospiraceae NK4A136 group, UCG‐005, Clostridium sensu stricto 1, Bacilli, Treponema, Monoglobus, Romboutsia, RF39, Prevotella, Oscillospiraceae, Turicibacter, [Eubacterium] coprostanoligenes group, Ruminococcaceae, and Roseburia* (Figure 5B).

IDD modeling experiments upregulated the relative abundance of *Muribaculaceae*. FMT down‐regulated this relative abundance. Next, we explored differences in community structure among the groups. The obtained value (R) = 0.263, and *P* = 0.001 indicated that there was a significant difference between the groups (Figure 5C). Non‐metric multidimensional analysis (NMDS) revealed differences among the control, IDD, and IDD + FMT groups (Figure 5D). Figure 5E shows the top 10 dominant flora with relative abundances at the genus level. IDD increased the abundance of *Lactobacillus* and decreased that of *ClostridiaUCG‐014*. FMT further increased the abundance of *Lactobacillus* and decreased that of *Clostridia_UCG‐014*. Thus, FMT increased the gut microbiota diversity and microbial abundance in rats treated with IDD.

## Discussion

IDD is a common musculoskeletal disorder that causes heavy socioeconomic burden.^31^, ^32^ Unfortunately, current diagnostic techniques and treatments do not reduce related pain.^33^, ^34^ Therefore, we attempted to treat the IDD rat model using FMT, and explore FMT's potential role in IDD. In this work, we studied the effects of FMT on histopathological damage, gut microbiota, inflammation, ECM homeostasis, and pyroptosis. We found that the expression of inflammatory factors (TNF‐α, IL‐1β, and IL‐6), catabolic enzyme (MMP‐3 and MMP‐13), and pyroptotic factors (NLRP3 and Caspase‐1) increased in the IDD group rats. In contrast, extracellular matrix (Collagen II and aggrecan) levels were downregulated. Additionally, vertebral disc tissue was severely damaged in the IDD group, with disordered cell arrangement and uneven safranin coloration. FMT reversed the effects of IDD modeling on these factors and alleviated cartilage tissue damage. Moreover, we also found that FMT increased the gut microbiota diversity and microbial abundance in rats with IDD.

### FMT Positively Affects the Maintenance of Vertebral Disc ECM Homeostasis

A key factor in IDD degeneration is how NPs sense and respond to the disease‐causing signals. For example, acid‐sensing ion channel (ASIC) 1/3 induces pyroptosis and NLRP3 inflammasome activation by upregulating ROS/NF‐κB signaling.^35^ We found that the levels of catabolic enzymes (matrix metalloproteinases [MMPs] ‐3 and MMP‐13) were upregulated and the levels of ECM (Collagen II and aggrecan) were downregulated in the vertebral disc tissue of IDD rats. FMT treatment reversed this trend. MMPs play an important role in mediating ECM disruption in cells.^36^ They degrade various ECM proteins.^37^ M2a macrophages promote ECM metabolic imbalance in NP cells, with enhanced transcription of Collagen II and aggrecan and decreased expression of MMP‐3 and MMP‐9.^38^ Three interacting events cause IDD degeneration: loss of NPs, ECM dehydration, and decreased cellular function.^11^, ^12^, ^13^, ^14^ The loss of localized cells was attributed to a reduction in matrix components, such as Collagen II, with a concomitant loss of water from hyaluronic acid aggregated into proteoglycans. The reduction in these components is associated with the replacement of the matrix by other fibers and proteoglycans.^39^, ^40^ Therefore, FMT positively affects the maintenance of vertebral disc ECM homeostasis.

### FMT Alleviated Inflammation and Pyroptosis in IDD Rats

In the IDD group, the expression of inflammatory factors (TNF‐α, IL‐1β, and IL‐6) and pyroptosis factors (NLRP3 and Caspase‐1) increased, and FMT reversed these changes. Previous studies have shown that the expression of TNF‐α, IL‐1β, and IL‐6 decreases when inflammation is decreased.^41^ Ethanol treatment of hepatocytes results in TXNIP overexpression, activation of the NLRP3 inflammasome, and Caspase‐1‐mediated pyroptosis.^42^ Pyroptosis is an inflammatory form of regulatory cell death (RCD).^43^ NLRP3‐mediated pyroptosis has been implicated in disc degeneration.^44^ NLRP3 inflammasome is thought to contribute to the induction of pyroptosis *via* TXNIP.^45^ The major destructive effect of pyroptosis depends on the release of inflammatory factors IL‐1β and IL‐18.^46^ When pattern recognition receptors recognize damage‐associated molecular patterns, the inflammasome activates Caspase‐1 followed by the activation of IL‐1β, IL‐18, and gasdermin‐D (GSDMD).^47^ Thus, FMT alleviated inflammation and pyroptosis in IDD rats.

### Changes in Gut Microbiota after FMT

Although fecal microbiota supernatants contain pro‐inflammatory metabolites, they can be removed by repeated washing.^48^ The synergistic action of phages and host bacteria after FMT restored the recipient gut microbiota.^49^ FMT prevented the decline in endogenous insulin production in patients newly diagnosed with T1D. Several microbiota are associated with preserved residual β‐cell function.^50^ We found that IDD increased the abundance of *Muribaculaceae* and *Lactobacillus*, and decreased the abundance of *Clostridia_UCG‐014*. FMT further increased the abundance of *Lactobacillus* and decreased that of *Clostridia_UCG‐014*. These studies demonstrate that IDD may have a potential therapeutic effect through the auxiliary metabolism of gut microbes.

### Limitations and Strengths of the Study

The advantage of this study were: (i) FMT maintained cellular stability, alleviated histopathological damage; and (ii) FMT influenced the gut microbiota in IDD rats. FMT may serve as a promising target for ameliorating IDD and for palliative therapy.

However, several limitations of this study must be considered. Owing to the limitations of experimental funds and conditions, we did not conduct experiments to explore the influence of specific components of fecal bacteria on IDD. In addition, the maintenance time of FMT treatment effect on IDD needs to be further explored.

### Conclusion

These findings suggest that FMT has a positive effect on maintaining cellular stability and alleviating histopathological damage. FMT decreased level of inflammatory factors, catabolic enzyme, and pyroptotic factors, and increased extracellular matrix levels. Besides, FMT alleviated the injury of intervertebral disc tissue in IDD rats. In addition, we found that FMT affected the gut microbiota diversity and microbial abundance in IDD rats. Therefore, FMT may serve as a promising target for ameliorating IDD and palliative therapy.

## Ethics Statement

All animal experiments were approved by the Animal Ethics Committee of Xiangya Third Hospital of Central South University (No.2020‐S292).

## Funding

This study was supported by the Hunan Health Commission's 2019 Medical Service and Support Capability Improvement Subsidy Fund (No. 2019‐57) and General Items of the Hunan Provincial Health Commission (No. 202204074627).

## Author Contributions

Ziwei Han and Li Wan conceived the design; Bo Yao, Youquan Cai, and Weiguo Wang carried out the experiment; Jia Deng, Lei Zhao, Ziwei Han, and Li Wan carried out the data visualization. Bo Yao wrote the first draft, and Ziwei Han and Li Wan directed the revisions. All authors have checked the final manuscript.
